# Teneurin-1 is expressed in interconnected regions of the developing brain and is processed in vivo

**DOI:** 10.1186/1471-213X-8-30

**Published:** 2008-03-25

**Authors:** Daniela Kenzelmann, Ruth Chiquet-Ehrismann, Nathaniel T Leachman, Richard P Tucker

**Affiliations:** 1 Friedrich Miescher Institute, Novartis Research Foundation, Maulbeerstr. 66, 4057 Basel, Switzerland; 2 School of Medicine, 1 Shields Avenue, University of California at Davis, Davis, CA 95616-8643, USA

## Abstract

**Background:**

Teneurins are a unique family of transmembrane proteins conserved from *C. elegans *and *D. melanogaster *to mammals. In vertebrates there are four paralogs (teneurin-1 to -4), all of which are expressed prominently in the developing central nervous system.

**Results:**

Analysis of teneurin-1 expression in the developing chick brain by in situ hybridization and immunohistochemistry defined a unique, distinct expression pattern in interconnected regions of the brain. Moreover we found complementary patterns of teneurin-1 and-2 expression in many parts of the brain, including the retina, optic tectum, olfactory bulb, and cerebellum as well as in brain nuclei involved in processing of sensory information. Based on these expression patterns, we suspect a role for teneurins in neuronal connectivity.

In contrast to the cell-surface staining of the antibody against the extracellular domain, an antibody recognizing the intracellular domain revealed nuclear staining in subpopulations of neurons and in undifferentiated mesenchyme. Western blot analysis of brain lysates showed the presence of N-terminal fragments of teneurin-1 containing the intracellular domain indicating that proteolytic processing occurs. Finally, the teneurin-1 intracellular domain was found to contain a nuclear localization signal, which is required for nuclear localization in transfected cells.

**Conclusion:**

Teneurin-1 and -2 are expressed by distinct interconnected populations of neurons in the developing central nervous system. Our data support the hypothesis that teneurins can be proteolytically processed leading to the release of the intracellular domain and its translocation to the nucleus.

## Background

Teneurins are a family of type II transmembrane proteins originally discovered as pair-rule gene products in *Drosophila *[1,2] and [3]. Homologs have been identified in chicken, mouse and humans called teneurin-1 to -4 (for reviews see [4] and [5]).

Teneurin domain architecture is highly conserved across phyla. All teneurins described to date have an extracellular domain (ECD) with eight tenascin-type EGF-like repeats followed by a region of conserved cysteines and YD-repeats [6]. The N-terminal intracellular domain (ICD) of vertebrate teneurins has a conserved and unique domain architecture, containing two polyproline motifs, EF-hand-like metal ion binding domains and several putative phosphorylation sites.

The expression of vertebrate teneurins has been studied most extensively in mouse and chicken. All of these reports are in agreement that the primary site of teneurin expression is the developing central nervous system ([7-13]), although teneurins are also expressed outside the nervous system at sites of pattern formation like the limb bud [14,15]. The expression of teneurins in specific subsets of neurons is conserved from *Drosophila *and *C. elegans *to vertebrates. For example, in the chicken visual system teneurin-1 transcripts are concentrated in the neurons of the tectofugal pathway, whereas mRNA encoding teneurin-2 is concentrated in the neurons of the thalamofugal pathway [16]. The pattern and timing of expression led to speculation that teneurins might have a role in synaptic connectivity. Further evidence for such a function is provided by the observation that teneurin-1 and -2 promote neurite outgrowth in vitro [7,8], (see also [17]) and in vivo [18], and by the fact that teneurins can homo- and heterodimerize through their EGF-like repeats [19]. Recently, the teneurin-4 gene was found in a screen for genes differentially regulated in emx-/- mice, and because of its intriguing expression pattern, in situ hybridization was performed for all four teneurin paralogs revealing gradients of expression and striking complementary expression in the mouse forebrain [13]. Interestingly, the human teneurin-1 gene is located on the X-chromosome in a region to which several X-linked mental retardation (XLMR) syndromes have been mapped [7]. The predominant neuronal expression and suggested function in brain development make teneurin-1 a promising candidate gene for XLMR.

The discovery of teneurins in *Drosophila *as the only pair-rule gene that is not a transcription factor suggested that teneurins – although transmembrane proteins – could also have a nuclear function [3]. Transfection of a full-length teneurin-2 reporter construct with a transcriptional activator attached to its N-terminus resulted in transcription of a reporter gene particularly in response to homotypic interactions, indicating that the ICD is released upon specific stimuli [20]. In vitro the teneurin-2 ICD localizes to PML-bodies in the nucleus and affects zic-1 mediated transcription [20]. Similarly, the teneurin-1 ICD colocalizes in the nucleus with MBD1, a methyl CpG binding protein, after co-tranfection of cells in culture [21]. Western blot analysis of cells transfected with full-length teneurin-1 show several N-terminal peptides which could correspond to intermediate processing products and the ICD, but it was not possible to visualize the ICD in the nucleus by immunofluorescence [21]. Assuming that the released intracellular domain has an important signalling function, it is likely that such a cleavage is tightly regulated during development and that it would not occur spontaneously in cell culture, or only to a very small extent.

The aim of this work was to analyze teneurin-1 expression in detail and to determine if teneurins can be processed in the vertebrate embryo as they appear to be processed in vitro. We have studied the expression pattern and processing of teneurin-1 in the developing chicken embryo using in situ hybridization, immunhistochemistry and immunoblotting, and have compared the patterns of teneurin-1 expression with those of teneurin-2. As antisera to teneurin-1 were raised against both the ICD and ECD of the protein, we were able to show for the first time the nuclear localization of the ICD in neurons and mesenchymal tissues in situ. The processing of teneurin-1 in developing tissues was confirmed by immunoblot analysis. Finally, we show that the teneurin-1 ICD contains a specific nuclear localization signal that is required for translocation into the nucleus.

## Results

### In situ localization of teneurin-1 transcripts

**Figure 1:**
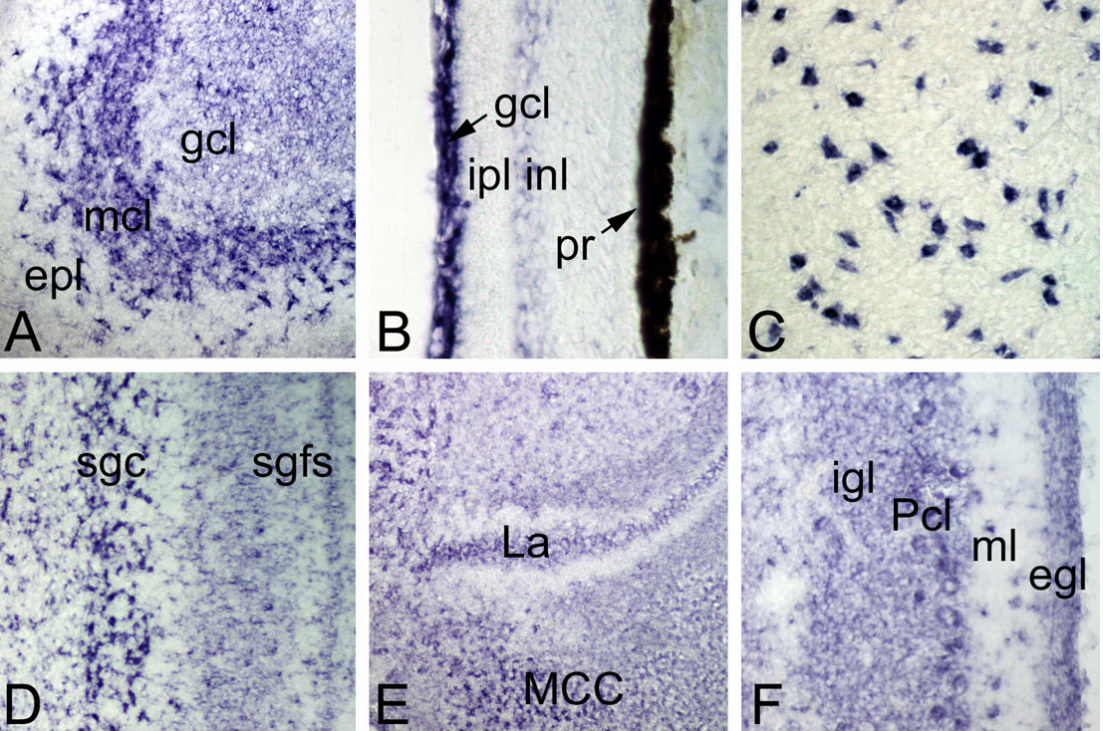
**Teneurin-1 is expressed in many parts of the developing CNS**. In situ hybridization at E17 with a teneurin-1 antisense probe (sense controls were negative). In the olfactory bulb (A), there is a strong hybridization signal in the mitral cell layer (mcl). The ganglion cell layer (gcl) is also positive, but the external plexiform layer (epl) is not. In the retina (B), the ganglion cell layer (gcl) is labelled intensely, and there is a faint signal in neurons of the inner nuclear layer (inl) adjacent to the inner plexiform layer (ipl). The pigment retina (pr) has dark melanosomes. The nucleus rotundus (C) contains large, scattered neurons that are positive for teneurin-1 mRNA. In the optic tectum (D) teneurin-1 mRNA is widespread, but is seen most clearly in the large neurons of the stratum griseum centrale (sgc). In the hindbrain (E) the nucleus laminaris (La) and nucleus magnocellularis (MCC) are labelled, as are Purkinje cells (Pcl) and other neurons in the cerebellum (F).

Teneurin-1 was widely expressed in the chick nervous system at embryonic day (E) 17. The strongest hybridization signals seen with the teneurin-1 antisense probe were in the mitral cells of the olfactory bulb (Fig. 1A), subpopulations of neurons in the hippocampus and piriform cortex (not shown, but see immunohistochemistry), retinal ganglion cells and cells found in the inner nuclear layer adjacent to the inner plexiform layer (Fig. 1B), the large neurons found within the rotund nucleus (Fig. 1C), and the neurons of the stratum griseum centrale of the optic tectum (Fig. 1D). In the hindbrain teneurin-1 mRNAs were found in the nucleus laminaris and nucleus magnocellularis (Fig. 1E) and throughout the cerebellum (Fig. 1F). Adjacent sections processed with a sense probe were unlabelled. Thus, the expression of teneurin-1 in the chicken embryo is more widespread than suggested by our earlier studies using a less sensitive radiolabeled probe that only detected the most abundant sites of expression in the visual system. In addition to the strong expression in interconnected subset of neurons in the tectofugal portions of the visual system (e.g., retinal ganglion cells, the stratum griseum centrale of the optic tectum and the rotund nucleus), teneurin-1 transcripts were also present in diverse brain regions involved in general sensation, olfaction, the processing of auditory information, and the coordination of complex motor behavior.

### Immunolocalization of teneurin-1 and teneurin-2

**Figure 2:**
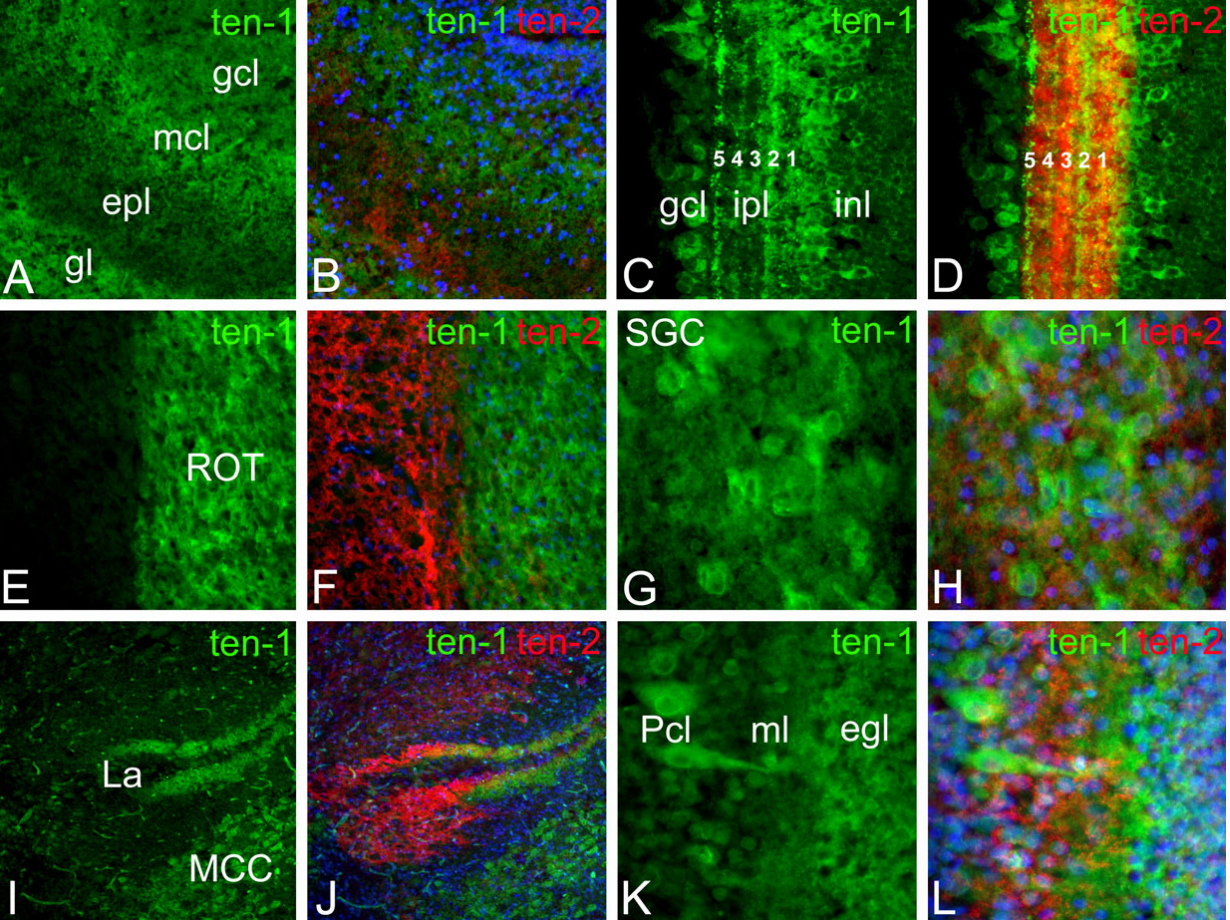
**Teneurin-1 is expressed in interconnected regions of the brain and is complementary to teneurin-2 expression**. Immunostaining of teneurin-1 and teneurin-2 in E17 brain. Green, teneurin-1; red, teneurin-2, blue (Hoechst), nuclei. In the olfactory bulb (A, B) the teneurin-1 antibody labels the mitral cell layer (mcl) and the glomerular layer. The external plexiform layer (epl) is positive for teneurin-2. In the temporal retina (C, D) subsets of neurons within the ganglion cell layer (gcl) and inner portion of the internal nuclear layer (inl) are positive for teneurin-1, as are laminae 2 and 5 within the inner plexiform layer (ipl). Teneurin-2 immunoreactivity is concentrated in laminae 1 and 3 of the ipl. The nucleus rotundus (E, ROT) is intensely labelled with teneurin-1 antibody, but not with anti-teneurin-2 (F). In the optic tectum (G, H) large neurons in the stratum griseum centrale (SGC) are positive for teneurin-1, but not teneurin-2. In the hindbrain (I, J), the nucleus laminaris (La) and nucleus magnocellularis (MCC) are labelled with the teneurin-1 antibody. Teneurin-1 and teneurin-2 immunoreactivity are found in distinctive dendritic fields within the La. In the cerebellum (K, L) teneurin-1 is found in Purkinje cells (Pcl) and in the molecular layer (ml) adjacent to the external granular layer (egl). In contrast, the anti-teneurin-2 labels the internal portion of the ml.

Polyclonal antibodies raised against recombinant fragments of teneurin-1 were used to study the distribution of teneurin-1 protein in the developing avian nervous system. Our results are summarized in Figure 2 and are briefly described here starting in the rostral forebrain and ending in the hindbrain, emphasizing regions also illustrated in Figure 1. At E17 the anti-teneurin-1 serum labelled the glomerular layer and mitral cell layer of the olfactory bulb (Fig. 2A). This pattern is consistent with the expression of teneurin-1 by mitral cells shown in Figure 1A and the transportation of the protein to the dendrites of mitral cells in the glomerular layer. In the retina teneurin-1 immunolabelling was observed in retinal ganglion cells, neurons within the inner nuclear layer adjacent to the inner plexiform layer, and within distinctive laminae (especially 2 and 5) within the inner plexiform layer (Fig. 2C). As predicted by in situ hybridization, teneurin-1 immunoreactivity was particularly strong throughout the rotund nucleus (Fig. 2E) and in the large neurons of the stratum griseum centrale of the optic tectum (Fig. 2G), two interconnected populations of neurons that make up part of the tectofugal visual pathway. Finally, the anti-teneurin-1 labelled the dendritic field of the external portion of the nucleus laminaris (Fig. 2I), the large neurons of the nucleus magnocellularis (Fig. 2I), and in the cerebellum labelled Purkinje cells and the portion of the molecular layer adjacent to the external granular layer (Fig. 2K). Note that the teneurin-1 expression pattern observed with the antibody is more extensive in most brain regions than observed by in situ hybridization. For example, neurons in the outer portion of the inner nuclear layer of the E17 retina are stained with the antibody (Fig. 2C), but are not positive for teneurin-1 transcripts (Fig. 1B). Since these cells were not stained with preimmune serum or secondary antibody only controls (not shown), a possible explanation for this discrepancy is that teneurin-1 transcripts are present below the level of detection of our in situ hybridization protocol in some regions, but nevertheless at levels sufficient to generate enough protein to be recognized, albeit faintly, by immunohistochemistry.

In situ hybridization has previously suggested that the patterns of expression of teneurin-1 and teneurin-2 are largely complimentary with the possible exception of the retina, where transcripts encoding these proteins appeared to co-localize in retinal ganglion cells and in cells near the developing inner plexiform layer, which may be amacrine cells. Here we were able to compare the distribution of teneurin-1 and teneurin-2 by superimposing the patterns of expression found on adjacent frozen sections stained with antisera specific for each protein. In each region examined, there was very little overlap in the expression of the two proteins. For example, in the olfactory bulb teneurin-2 immunoreactivity was seen in the external plexiform layer sandwiched between the layers of teneurin-1 expression (Fig. 2B). In the retina both teneurin-1 and teneurin-2 were found in the inner plexiform layer, but they are concentrated in different laminae: teneurin-1 is found primarily in laminae 2 and 5, while teneurin-2 is most prominently expressed in laminae 1 and 3 (Fig. 2D). In the thalamus teneurin-2 immunoreactivity was seen in the pretectal region and was completely excluded from the adjacent rotund nucleus (Fig. 2F). In the optic tectum anti-teneurin-2 immunolabelled puncta surrounding the large neurons of the stratum griseum centrale, but did not label the neuronal cell bodies themselves (Fig. 2H). The complementary expression of the two teneurins was particularly evident in the nucleus laminaris, where teneurin-2 immunoreactivity was seen in the thick dendritic field of the internal portion of the lamina and teneurin-1 was found in the thinner external portion (Fig. 2J). Finally, while teneurin-1 and teneurin-2 are both found in the molecular layer of the developing cerebellum, teneurin-1 was found in the younger, external portion of this layer whereas teneurin-2 was found in the more mature, internal portion (Fig. 2L). A summary of teneurin-1 and -2 expression in the developing chick brain is provided in Table 1.

**Table 1:** The expression of teneurin-1 and teneurin-2 in the E17 avian central nervous system

Region	Teneurin-1 Intensity*	Teneurin-2 Intensity
Olfactory bulb: Glomerular layer	++	-
Olfactory bulb: External plexiform layer	-	+
Hippocampus	+/-	+++
Hyperstriatrum accessorium	-	++
Hyperstriatum ventrale	-	++
Nucleus septalis lateralis	-	++
Nucleus taeniae	-	++
Posteromedial cortex piriformis	+/-	+++
Nucleus dorsolateral anterior thalami	-	++
Nucleus geniculatus lateralis	-	++
Nucleus pretectalis	-	++
Nucleus rotundus	+++	-
Nucleus spiriformis	-	++
Nucleus triangularis	-	++
Retina: Ganglion cell layer	+/-	-
Retina: Inner plexiform layer 5	+	-
Retina: Inner plexiform layer 4	-	+/-
Retina: Inner plexiform layer 3	-	++
Retina: Inner plexiform layer 2	+	-
Retina: Inner plexiform layer 1	-	+
Retina: Inner nuclear layer	+/-	-
Retina: Outer plexiform layer	-	+/-
Nucleus isthmi, pars magnocellularis	-	++
Nucleus isthmi, pars parvocellularis	-	++
Nucleus isthmo-opticus	-	++
Nucleus lentiformis mesencephali	-	++
Nucleus of Edinger-Westphal	-	++
Stratum griseum centrale	++	-
Stratum griseum et fibrosum superficiale	+/-	+
Stratum griseum periventriculare	-	+
Nucleus laminaris	++	+/-
Nucleus magnocellularis	++	-
Cerebellum: Molecular layer	+/-	+/-
Cerebellum: Purkinje cell layer	+	-
Cerebellum: Granule cell layer	-	+/-

*+++, intense signal, visible at low magnification; ++, strong signal, visible at intermediate magnification; +, weak signal, visible at high magnification; +/-, weak and sometimes missing; -, no signal

### Nuclear localization of the intracellular domain in situ

**Figure 3:**
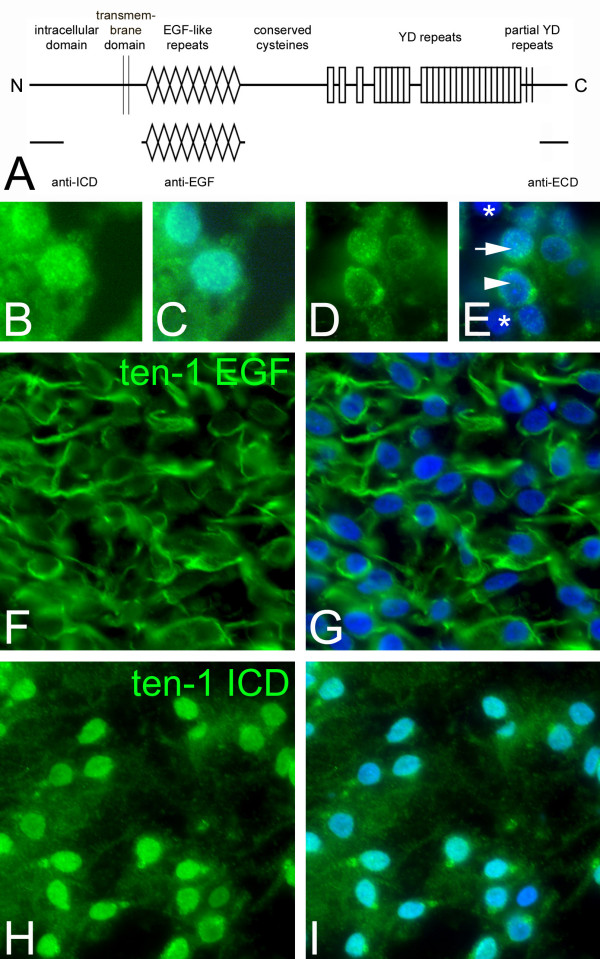
**Nuclear teneurin-1 intracellular domain is observed in subsets of neurons and in specific tissues**. Schematic diagram of teneurin domain architecture and antibodies used in this study (A). Comparison of immunostaining with antibodies to the extracellular and intracellular domains in sections and in vitro (B to I). Neurons of the piriform cortex at E17 (B, C) and neurons found in the retinal ganglion cell layer at E12 (D, E) stained with anti-ICD. The anti-ICD labels nuclei in the piriform cortex and labels a subset of retinal ganglion cells (arrow), but other TuJ1-positive neurons (label not shown) in the retinal ganglion cell layer are labeled with anti-ICD teneurin-1 but do not exhibit nuclear staining (arrowhead). There are also TuJ1-positive neurons in the retinal ganglion cell layer that are negative for teneurin-1 (asterisk). Head mesenchyme at E7 stained with anti-EGF (F, G) and anti-ICD (H, I). In the head mesenchyme, the anti-ICD labels the nucleus of most cells, whereas the anti-EGF exhibits cell-surface staining. Green, teneurin-1; blue (Hoechst), nuclei.

Antibodies detecting the ICD (anti-ICD) or the ECD (anti-EGF and anti-ECD, see Fig. 3A) of teneurin-1 were used to detect teneurin-1 expression in brain homogenates harvested from chicken embryos.

The ICD antibody was used to determine if the ICD of teneurin-1 can be detected in the nucleus in situ with immunohistochemistry. We found nuclear teneurin-1 ICD in a few regions of the developing brain. One region where the majority of neurons expressing teneurin-1 showed nuclear localization with the anti-ICD was the E17 piriform cortex (Fig. 3B, C). Here the anti-ICD stained fine puncta within nuclei as determined by co-staining with the Hoechst nuclear stain. The same fine puncta were labelled in the nuclei of neurons within the retinal ganglion cell layer at E12 (Fig. 3D, E). However, unlike the labelling of the piriform cortex the labelling pattern within the retinal ganglion cell layer was diverse: the nuclei of some cells are labelled (arrow, Fig. 3E), in some cells only the cytoplasm or cell membrane was labelled (arrowhead, Fig. 3E), and other cells were completely unlabelled (asterisks, Fig. 3E). All of these cells were identified as neurons by double labelling with the neuronal marker TuJ1 (not shown). The appearance of the ICD of teneurin-1 in the nuclei of retinal neurons is temporally and spatially regulated, as nuclear localization in the retinal ganglion cell layer was not observed at E7 or E17 (not shown), nor was it observed in neurons in the inner nuclear layer at any of the time points used in this study. The most dramatic examples of nuclear localization of the ICD of teneurin-1 were seen in non-neuronal tissues. In patches of head mesenchyme of E7 and E12 embryos the anti-ICD labelled the nuclei of fibroblasts intensely (Fig. 3H, I), in contrast to the antibody raised against the EGF-like repeats found in the ECD (Fig. 3F, G). The nuclear staining was specific, as it was abolished by preabsorbing the antibody with the ICD-peptide (not shown). Note that these two antibodies (anti-ICD and anti-EGF) stained the same regions of the fore, mid and hindbrain identically except for the occasional staining of some cell nuclei by the former and not the latter. Also, the anti-ICD stained some endothelial cells and the anti-EGF did not (e.g., note the endothelial cell labelling in Fig. 2I, which was stained with anti-ICD).

### Teneurin-1 appears to be proteolytically processed

**Figure 4:**
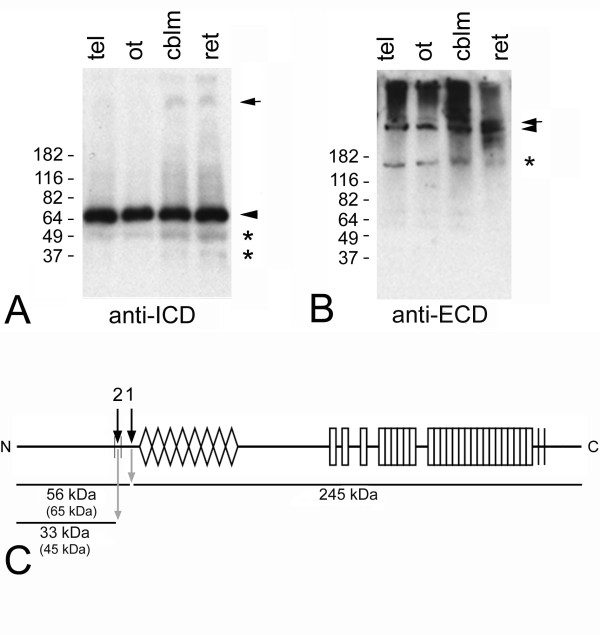
**Teneurin-1 appears to be processed in the developing chicken brain**. Western blot analysis of embryonic chicken brain lysates. Detection of endogenous teneurin-1 using anti-ICD (A) and anti-ECD antibodies (B). The numbers to the left indicate the positions of molecular weight standards. The anti-ICD detects a prominent N-terminal fragment at 65 kDa (arrowhead), which is much smaller than expected for the full-length protein (arrow), suggesting proteolytic processing. In contrast, using the anti-ECD antibody results in detection of the full-length protein (arrow) and a large C-terminal fragment (arrowhead). Asterisks label additional processing products (see text for details). Postulated cleavage sites and calculated molecular weights of processing products (C). Postulated cleavage sites: 1 = ectodomain shedding, 2 = intramembrane cleavage releasing the ICD. The sizes indicated are predicted from the amino acid sequence; known apparent molecular weights are shown in parentheses (see text for details). The calculated molecular weights are approximate, as the exact cleavage sites are not known.

Western blot analysis using the ICD antibody showed a prominent band at 65 kDa (Fig. 4A, arrowhead), which is much smaller than expected for the full length protein (> 300 kDa). The specificity was confirmed by competition with the ICD peptide which completely abrogates detection of this band (not shown). However, 65 kDa is larger than we would expect for the teneurin-1 ICD, because an N-terminal fragment containing the amino acids up to the transmembrane domain (ICD construct) migrates at 45 kDa [21]. Therefore, this fragment may still contain the transmembrane domain. Such an N-terminal fragment would be expected to occur after ectodomain shedding (Fig. 4C, cleavage 1). The full-length protein was barely detectable (Fig. 4A, arrow), indicating that ectodomain shedding may take place constitutively. The faint bands observed below the 65 kDa band (Fig. 4A, asterisks) at approximately 50 and 35 kDa might be the released ICD generated after the second cleavage (Fig. 4C, cleavage 2), which occurs only in a specific subset of cells. These data suggest that proteolytic processing of the full-length protein takes place in vivo, resulting in N-terminal fragments. When the same extracts were analyzed using an antibody detecting the ECD (Fig. 4B), a faint band > 250 kDa (arrow) and a stronger band around 250 kDa (arrowhead) are observed. Since the predicted molecular weight of full-length teneurins is more than 300 kDa, the upper band may represent the full-length protein, and the lower band could correspond to the ECD after shedding, which has a predicted molecular weight of 245 kDa (Fig. 4C). The smear above these bands can be explained by glycosylation of the extracellular part of the protein [7]. The band at 150 kDa (Fig. 4B, asterisk) could be a further cleaved extracellular fragment of teneurin-1. In Figure 4C the postulated cleavage sites and the resulting fragments are summarized. Note that the molecular weights are either calculated based on the amino sequences or based on observations (shown in parentheses). The ICD has a higher apparent molecular weight than predicted, therefore we expect the same to be the case for the 65 kDa N-terminal fragment, for which the predicted molecular weight would only be 56 kDa (Fig. 4C).

### Identification of a nuclear localization signal

**Figure 5:**
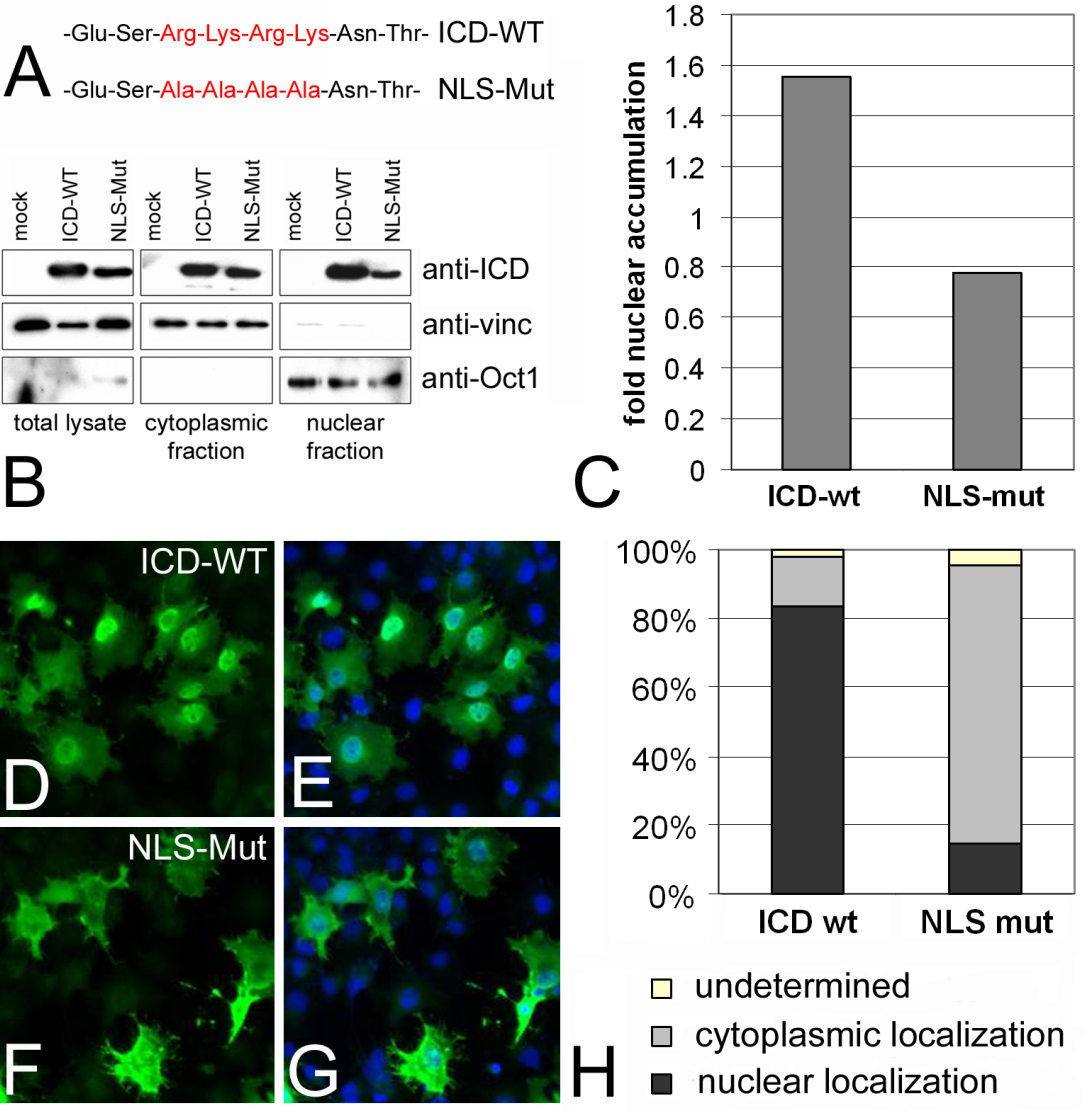
**The teneurin-1 intracellular domain contains a nuclear localization signal**. The amino acid sequence representing a putative nuclear localization signal (NLS) which was mutagenized to alanines (A). Western blot analysis of total lysates, cytoplasmic and nuclear fractions of COS-7 cells mock transfected, transfected with wild type ICD (ICD-WT) or transfected with the mutated NLS (NLS-Mut) constructs (B). Blots were probed with the anti-ICD, an antibody to the cytoplasmic protein vinculin, or an antibody to the nuclear protein Oct-1. Western blot quantification and calculation of the nuclear-to-cytoplasmic ICD ratio (C). Immunofluorescence staining of transfected COS-7 cells (D-G) and quantification of nuclear localization by cell counting (H). Both western blot analysis and immunofluorescence show that the overexpressed wild-type ICD is localized in the nucleus, whereas the NLS-Mut construct is predominantly found in the cytoplasm.

The ICD of teneurin-1 contains the amino acid sequence arginine-lysine-arginine-lysine at position 62 to 65 from the N-terminus. This resembles a classical nuclear localization signal (NLS), which directs the nuclear import of proteins. This putative NLS is conserved in chicken, mouse and human teneurin-1. To test whether it is functional, the four basic amino acids were replaced by alanines in the construct named NLS-mut (Fig. 5A). COS-7 cells were transfected either with a construct encoding the ICD of teneurin-1 (ICD-wt) or the NLS-mut construct. Western blot analysis of cytoplasmic and nuclear extracts of the transfected cells showed decreased nuclear accumulation of the NLS-mut construct compared to the ICD-wt construct (Fig. 5B). Quantification of the nuclear-to-cytoplasmic ratio showed that ICD-wt accumulates in the nucleus, which is not the case for NLS-mut (Fig. 5C). The ICD-wt protein is detected in the nucleus by immunofluorescence (Fig. 5D, E). However, nuclear localization of the ICD was abolished if the NLS is mutated (Fig. 5F, G). More than 80% of the cells expressing the wt-construct exhibited nuclear staining, whereas this was only observed in 20% of the cells expressing the NLS-mut version (Fig. 5H). In addition to the strongly decreased nuclear localization, these cells also exhibited a more pronounced localization of the teneurin-1 ICD to the cell membrane and cell processes.

## Discussion

In this work we analyzed the expression of teneurin-1 in the chicken embryo by in situ hybridization and immunohistochemistry, focusing on the developing nervous system. Using an antibody recognizing the N-terminus of teneurin-1 we demonstrated for the first time the nuclear localization of a teneurin ICD in a developing vertebrate. This finding was supported by western-blot analysis of endogenous teneurin-1 and can be explained by the fact that the ICD contains a functional NLS. Thus, teneurin-1 is a transmembrane protein that can be processed to deliver a signal to the nucleus with the potential to alter gene expression.

### Teneurin-1 expression suggests a role in the establishment of neuronal connectivity

Teneurin-1 has a distinctive temporal-spatial expression pattern in the developing nervous system that includes interconnected regions in the brain. For example, in the tectofugal pathway of the avian visual system, retinal ganglion cells project to neurons in the stratum griseum centrale of the optic tectum, which in turn, project to the nucleus rotundus. Teneurin-1 is expressed prominently by each component of this pathway. However, teneurin-1 expression is not limited to the visual system, but is also present in other parts of the brain. In the olfactory pathway, teneurin-1 is expressed both in the olfactory bulb and the piriform cortex, which receives input from mitral/tufted cells of the olfactory bulb and is involved in processing of odor information. Teneurin-1 mRNA and immunostaining were also present in nuclei processing auditory information such as the nucleus laminaris and the nucleus magnocellularis. In previous studies of teneurin-1 expression, which relied on low resolution in situ hybridization techniques, the expression of teneurin-1 outside the avian visual system was not observed [7,8]. The broader expression reported here is in line with studies in mice, where teneurin-1 has been reported to be expressed in specific parts of the cortex [13] as well as the hippocampus and cerebellum [22,12]. In both mouse and chicken, a common feature is the expression of teneurin-1 by interconnected neurons (see review [5]).

One of the most interesting features of teneurins is the complementarity of their expression patterns. A previous study [16] reported the presence of teneurin-2 in the thalamofugal visual pathway; here we confirmed the expression of teneurin-1 in the tectofugal visual pathway. This complementarity is again not limited to the visual system, but appears also in the olfactory bulb and in the nucleus laminaris and nucleus magnocellularis. The most intriguing complementary pattern was found in the inner plexiform layer in the retina, which is subdivided in five laminae. There, axons of the amacrine and bipolar cells meet the dendrites of retinal ganglion cells. Because most of these nerve terminals are confined to distinct laminae, it is thought that lamination is a major determinant of synaptic specificity in the retina. Teneurin-1 and teneurin-2 were found in distinct, non-overlapping laminae, suggesting that they are expressed by distinctive subpopulations of neurons. Other examples of molecules present in distinct sublaminae of the retina were reported by Yamagata et al., including the adhesive proteins sidekick-1 and -2 [23]. Retinal ganglion cells expressing sidekick-1 or -2 preferentially arborize in sidekick-1 or-2 positive sublaminae, and ectopic expression of each sidekick directs dendrites to sublaminae where the corresponding sidekick is present [24]. These observations suggest roles for each of the teneurins in the development of different neuronal pathways. The current study also provides evidence that a given neuron may switch teneurin expression as development proceeds. In the cerebellum, for example, teneurin-1 immunoreactivity is seen in the axons of granule cells adjacent to the external granular layer, whereas teneurin-2 immunoreactivity is seen in the axons of the older granule cells near the perikarya of Purkinje cells.

Based on its expression pattern we suggest a role for teneurins in the establishment of appropriate connectivity in the developing brain. This task is preformed in several steps, including the generation of appropriate neuronal cell types, migration of neurons to specific nuclei or laminae, growth of axons to target areas, and the formation and maintenance of synapses within the target [25-27]. Recent evidence suggests that the same molecules can be involved in several of these steps [28]. Currently there is no knowledge about a possible role of teneurins in defining neuronal precursor subpopulations, but there are some data indicating that teneurins are involved in the outgrowth and migration of axons. For example, teneurins promote neurite outgrowth in vitro, and recently published in vivo data show that the cell bodies of neurons expressing teneurin-3 (tenm_3) tend to cluster, their processes are intertwined, and have increased neurite outgrowth [18].

In 1963, Roger Sperry formulated his chemoaffinity hypothesis, according to which growing axons carry biochemical "tags" which allow them to find their appropriate target region [29]. In the meantime, many molecules that act in such a manner have been discovered; the best known example is the cadherin superfamily. Cadherins not only act as cell adhesion molecules through homophilic interactions mediated by their ECD, but are also involved in signal transduction through their ICDs. Many cadherins are also expressed in very restricted regions in the brain indicating a role in establishment of neuronal connections [30,31]. The observation that teneurins can mediate homophilic and heterophilic interactions suggests that they might function in a similar way to cadherins [19].

Regarding the complexity of the vertebrate brain, it is unlikely that only a few molecules govern compartmentalization and wiring in the developing brain. More probably, concerted action of many different genes is required, and it will be a challenge to define the exact role of teneurins within this genetic network. To date, very little is known about the factors which regulate teneurin expression and how the teneurins function. For further analysis of the role of teneurins in brain development, it will be crucial to manipulate the expression or function of teneurins in vivo.

### Teneurins appear to be processed in vivo, possibly by regulated intramembrane proteolysis

At specific sites we observed nuclear staining with the antibody to the ICD of teneurin-1. These sites, both neuronal and non-neuronal, were subsets of the regions where teneurin-1 was expressed. This was confirmed with in situ hybridization and with antibodies to the ECD of teneurin-1. Western blot analysis confirmed the presence of N-terminal fragments of teneurin-1. Additionally, the ICD was found to contain a conserved NLS, which we demonstrated was functional in cultured cells.

Regulated intramembrane proteolysis (RIP) may provide an explanation for the detection of a transmembrane protein like teneurin-1 in the nucleus. There is only a limited set of known intramembrane proteases, all of which exhibit specificity for either Type-I (presenilin, rhomboid) or Type-II (site-2 protease, signal peptide peptidase) transmembrane proteins. Therefore, only the latter two are possible candidates for teneurin processing, including the recently identified SPP-like proteases [32]. Site-2 protease was the first intramembrane protease discovered, releasing the transcription factor SREBP from the ER-membrane in response to sterol depletion [33]. In addition, it has been shown to cleave ATF6, another transcription factor, in response to ER stress [34]. The expression pattern of SPP studied in adult mice partially overlaps with teneurin expression in distinct brain regions [35]. No data are available of SPP-like proteases in vertebrates, but a study in *C. elegans *suggests an important role in development [36]. Presenilin is the most studied intramembrane protease, responsible for the cleavage of Notch [37-39], APP [40,41] and other substrates, including some cadherins and ephrins. Most C-terminal fragments resulting from presenilin processing exhibit signaling activity and many of them localize to the nucleus, where they regulate transcription [42]. However, since presenilin only cuts type-I transmembrane proteins, it is not a candidate protease for teneurin processing.

Western blot analysis showed a prominent band at about 65 kDa using the ICD-antibody, which is larger than the predicted size of the ICD. A common feature of RIP is that a cleavage on the extracellular side has to occur first, resulting in the release of the ECD, before the remainder of the protein can be a substrate for intramembrane cleavage. Therefore, this band most likely represents teneurin-1 after ectodomain shedding, which then, depending on the cellular context, is further processed to result in a soluble ICD (i.e., the faint smaller bands observed on the immunoblot). This fits with the observation that the 65 kDa band is very abundant in brain lysates, but nuclear ICD is observed in only few cells.

Based on the presence of nuclear ICD in mesenchyme as well as in a restricted subset of neurons, we can not draw a conclusion regarding a general biological function of teneurin processing during development. The occurrence of the nuclear ICD in such different cells and tissues implies a cell-type- or tissue-specific role for teneurin-1 intracellular signalling. This was particularly striking in the retina where we observed retinal ganglion cells negative for teneurin-1 and teneurin-1-positive cells with and without the nuclear ICD next to each other. One example of how to further address the biological relevance of these observations is provided by the elegant studies of Notch processing in genetically engineered mice. Replacement of the Notch ICD with Cre recombinase permitted the identification of cells in which Notch is cleaved, since the released Cre induces β-gal expression and labels specifically these cells [43].

Future work will have to be dedicated to finding the proteases responsible for teneurin processing and the elucidation of the function of the teneurin-1 ICD in the nucleus. Since this cleavage occurs only in subsets of cells at specific time points during development, it will be interesting to know if homophilic interactions or interactions with a yet to be identified ligand triggers the cleavage of the ICD.

## Conclusion

**Figure 6:**
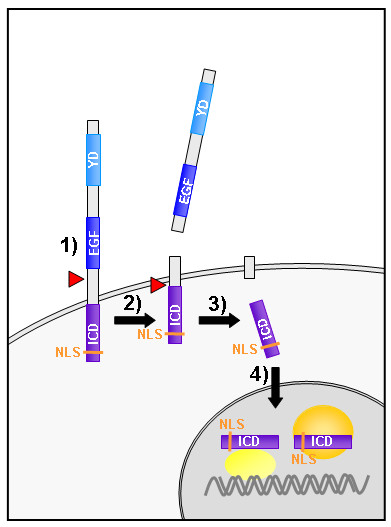
**Model of teneurin signalling**. This model describes how an intracellular domain (ICD) could be generated from a transmembrane protein and transported to the nucleus. This process takes place only in specific cells and tissues where the anti-ICD labeled nuclei. 1) A first protease sheds the extracellular domain (ECD) of teneurins. 2) The remainder of teneurin-1 can now become a substrate for an intramembrane protease. 3) The ICD is released. 4) The soluble ICD translocates to the nucleus via nuclear localization signal (NLS). *EGF, EGF-like repeats; YD, YD-repeats. Red arrows indicate postulated cleavage sites and the yellow spheres proteins interacting with teneurin-1 ICD in the nucleus*.

In this study we show that teneurin-1 is expressed in interconnected regions of the developing chick brain, and its expression pattern is complementary to teneurin-2. We present the first evidence of teneurin processing in vertebrates and demonstrate that the ICD of teneurin-1 contains a functional NLS. Our results provide support for the hypothesis that teneurins can be proteolytically processed to generate a soluble ICD that translocates to the nucleus (Fig. 6).

## Methods

### In situ hybridization

Chicken embryos were sacrificed at different developmental stages and fixed in cold 4% paraformaldehyde in phosphate buffered saline (PBS) for a few hours or overnight, depending on the age. For E17, brain and eyes were dissected out, otherwise whole embryos or heads were used. Tissues were cryoprotected in 25% sucrose overnight before embedding in OCT Tissue-Tek (Sakura). Horizontal, sagittal and transversal sections were cut on a Microm cryostat, mounted on Superfrost slides (Menzel-Gläser), dried and stored at -80°C. The probe was synthesized by in vitro transcription of a chicken teneurin-1 EcoRI-XbaI fragment corresponding to 628 bp within the ICD cloned into pSPT18 using the Roche DIG RNA labeling kit. In situ hybridization was performed on a Ventana automatic staining module and the Ventana RiboMap and BlueMap kits. The in situ hybridization protocol included prefixation, cell conditioning and protease pretreatment. Probe was allowed to hybridize for 6 h at 65°C followed by three washes with increasing stringency (1 × SSC, 0.5 × SSC and 0.1 × SSC at 65°C, respectively). After post-fixation, the probe was detected with anti-DIG (Roche) for 32 min and the slides were incubated with AP-substrate for 6 h. Antisense and sense controls were always performed in parallel.

### Immunohistochemistry

Cryosections of chicken embryos at various embryonic stages were prepared as outlined above. Sections were hydrated in PBS, blocked with 0.5% BSA and incubated overnight in appropriately diluted primary antibody. Primary antibodies included rabbit polyclonal antisera raised against recombinant proteins corresponding to: 1) the 160 N-terminal amino acids of the ICD of chicken teneurin-1 (anti-ICD, [21]; the 300 C-terminal amino acids of the extracellular domain of chicken teneurin-1 (anti-ECD, [21]; 3) a fusion of the N-terminus of tenascin-C (for protein purification purposes) and the 8 EGF-like domains of chicken teneurin-1 (anti-EGF; see Fig. 3A for a summary of the teneurin-1 antisera). The latter antiserum was preabsorbed against purified tenascin-C prior to use to eliminate background. A previously described antiserum to chicken teneurin-2 [8] and a commercially available neuronal marker (the mouse monoclonal antibody TuJ1; Sigma) were also used. After incubation overnight in primary antibody the sections were rinsed in PBS, blocked again in PBS/BSA, and incubated for 2 hr in Alexa-tagged anti-rabbit or anti-mouse secondary antibodies (Invitrogen). For controls, adjacent sections were incubated in either diluted preimmune serum, with secondary antibody alone or the primary antibody blocked with the antigen peptide. In all cases the control sections were unstained. Sections were viewed using Nikon Optiphot and a Zeiss Axiophot photomicroscopes with epi-illumination.

### Cloning of constructs, cell culture and transfection

The teneurin-1 ICD missing the NLS was produced by two-step PCR from pCEP-ten1 ICD. Primers containing codons for alanines instead of arginines and lysines were used to introduce the mutation. The second PCR product was cloned into pCEP using NotI and XbaI. Primers used for mutagenesis were pCEP fw: 5'AGAGCTCGTTTAGTGAACCT3', Ten1 NLS fw: 5'TACAACAGTCAAAGCGCGGCGGCGGCGAATACTGACCAATCC3', Ten1 NLS rev: 5'GGATTGGTCAGTATTCGCCGCCGCCGCGCTTTGACTGTTGTA3' and EBV rev: 5'GTGGTTTGTCCAAACTCATC3'.

To generate a fusion protein containing the tenascin-C N-terminus for secretion and the teneurin-1 EGF-like repeats for antibody-generation, two step PCR was performed using the following primers: KS:5' CGAGGTCGACGGTATCG3', TNC/EGF fw 5'AAAGGCCCCACCTGCTCCTTGTTTGGTTTAACCTGG3', TNC/EGF rev: 5'CCAGGTTAAACCAAACAAGGAGCAGTTGGGGCCTTT3' and EGF-His-Xho-rev: 5'ACTACTCGAGTTAGTGGTGGTGGTGGTGGTGCATTTCCATGACAACGTT3'. The PCR fragment was cloned using HindIII and XhoI into pCEP. This recombinant protein was expressed in 293T EBNA-cells and purified using the C-terminal HIS-tag to immunize rabbits. To remove tenascin-C immunoreactivity of the resulting antiserum, it was purified on a tenascin-C column.

COS-7 cells were maintained in DMEM containing 10% FCS. For transfection, the FUGENE reagent (Roche) was used according to the manufacturer's protocol. The cells were harvested 24 h after transfection for analysis.

### Immunocytochemistry following transfection

24 h after transfection, the cells were fixed for 10 min with 4% paraformaldehyde, permeabilized with 1% Triton-X, blocked with BSA for 15 min, and stained with anti-ICD (1:300) or anti-Flag (1:1000). Secondary antibodies coupled with FITC from Alexa were used, and the nuclei were counterstained with Hoechst dye. Images were acquired on a Z1 fluorescence microscope. The 40× objective was used to obtain images showing the localization of the teneurin-1 ICD in detail. Low-magnification pictures were used for quantification by counting cells and classifying them as nuclear, cytoplasmic or undetermined localization of the ICD. The experiment was repeated three times, capturing five random images per transfection each time. All transfected cells on the images were counted: 492 for the ICD- wt construct and 420 for the NLS-mut construct. Nuclear staining was classified when the staining intensity was higher in the nucleus than in the cytoplasm, otherwise the cells were scored as cytoplasmic. The scoring was performed by an independent person who was not aware of which images were from ICD-wt or NLS-mut transfections. Cells were classified as undetermined if they were dividing, or if the staining intensity was too bright to determine if it is nuclear or cytoplasmic.

### Western blot analysis

The specificity of the teneurin-1 and -2 antibodies was confirmed by detection of teneurin-1 and -2 in transfected cells. Chicken embryos at E17 were sacrificed and tissues were dissected out and frozen in liquid nitrogen. After grinding with a mortar and pestle, the powder was dissolved in RIPA buffer. Sample buffer containing β-mercaptoethanol was added and the lysate was heated at 95°C before loading on a 4–12% gradient gel (BioRad) for SDS-PAGE. The Benchmark prestained protein ladder and HiMark prestained protein standards (both from Invitrogen) were used to determine the size of the bands. Transfer to an immobilon membrane was performed at 45 V per gel for 2 h. For detection, both rabbit polyclonal antibodies anti-ICD and anti-ECD were used at a dilution of 1:1000.

To analyze COS-7 cell lysates, culture medium was removed and the cells were washed with PBS. For the total lysate, cells were lysed in sample buffer containing β-mercaptoethanol directly. The cytoplasmic and nuclear extract was prepared using the NuCLEAR kit from Sigma following the manufacturer's instructions. SDS-PAGE was performed on 12.5% gels, and the proteins transferred for 1 h at 45 V per gel. The transfected ICD was detected with an anti-ICD or anti-Flag antibody diluted 1:1000. Anti-vinculin (hvin1, Sigma, 1:1000) served as a cytoplasmic marker and oct-1 (Santa Cruz, 1:1000) as a nuclear marker. SuperSignal (Pierce) was used for detection of horseradish-peroxidase coupled secondary antibody, which was used at 1:10,000. Chemiluminescence detection films were exposed and developed, and the results were scanned for image processing. Quantification of the western blot was performed using ImageJ 1.33 [44]. The intensity of the entire bands was measured, and the background was substracted. The nuclear-to-cytoplasmic ration was calculated by dividing the nuclear intensity by the cytoplasmic intensity, which indicates nuclear accumulation of the ICD.

## Authors' contributions

DK carried out the in situ hybridization, western blot analysis and cell culture experiments and prepared the first draft of the manuscript. RC participated in planning and discussing the experiments and helped to write the manuscript. NL and RPT performed the immunohistochemistry, and RPT participated in writing the manuscript and preparing the figures. All authors read and approved the final manuscript.
